# Dissociable Neural Response Signatures for Slow Amplitude and Frequency Modulation in Human Auditory Cortex

**DOI:** 10.1371/journal.pone.0078758

**Published:** 2013-10-29

**Authors:** Molly J. Henry, Jonas Obleser

**Affiliations:** Max Planck Research Group “Auditory Cognition”, Max Planck Institute for Human Cognitive and Brain Sciences, Leipzig, Germany; University of Salamanca- Institute for Neuroscience of Castille and Leon and Medical School, Spain

## Abstract

Natural auditory stimuli are characterized by slow fluctuations in amplitude and frequency. However, the degree to which the neural responses to slow amplitude modulation (AM) and frequency modulation (FM) are capable of conveying independent time-varying information, particularly with respect to speech communication, is unclear. In the current electroencephalography (EEG) study, participants listened to amplitude- and frequency-modulated narrow-band noises with a 3-Hz modulation rate, and the resulting neural responses were compared. Spectral analyses revealed similar spectral amplitude peaks for AM and FM at the stimulation frequency (3 Hz), but amplitude at the second harmonic frequency (6 Hz) was much higher for FM than for AM. Moreover, the phase delay of neural responses with respect to the full-band stimulus envelope was shorter for FM than for AM. Finally, the critical analysis involved classification of single trials as being in response to either AM or FM based on either phase or amplitude information. Time-varying phase, but not amplitude, was sufficient to accurately classify AM and FM stimuli based on single-trial neural responses. Taken together, the current results support the dissociable nature of cortical signatures of slow AM and FM. These cortical signatures potentially provide an efficient means to dissect simultaneously communicated slow temporal and spectral information in acoustic communication signals.

## Introduction

Natural auditory stimuli, including speech and non-human animal vocalizations, are characterized by slow fluctuations in amplitude and frequency. For example, human speech contains amplitude variations corresponding to the syllable envelope (~2–7 Hz; [1-3]) and slower frequency variations corresponding to prosodic contour (1–3 Hz; [4]). An important research question concerns the degree to which the time-varying neural signatures of amplitude modulation (AM) and frequency modulation (FM) differ, and thus the extent to which the two modulation types are capable of communicating independent “streams” of information. In this respect, there are (at least) two levels of analysis that can be considered with respect to the nature of AM and FM processing. Peripheral coding of AM and FM has been studied extensively using psychophysical paradigms; below, we will briefly review ideas stemming from an “excitation pattern” hypothesis, which describes peripheral modulation encoding in terms of the responses of frequency-tuned cochlear filters. Cortical modulation coding has been previously studied in the context of invasive animal recordings and at mostly high modulation rates using human electro- and magnetoencephalography (EEG/MEG). The current study focuses on the time-varying cortical representations of AM and FM, specifically in the context of slow, speech-relevant modulation rates. In particular, we directly compared the amplitude and phase characteristics of EEG responses to slow (3-Hz) AM and FM in order to characterize the features of the cortical response that would afford potential perceptual separation of the two modulation types. In particular, we used a single-trial classification approach that involved categorization of neural responses based on phase or amplitude information. 

### Peripheral encoding of AM and FM

With respect to the peripheral encoding of temporal modulation, an excitation pattern hypothesis describes AM and FM encoding in terms of the corresponding time-varying cochlear-filter output [5-7]. Consider neural responses to AM and FM beginning at the tonotopically-organized periphery of the auditory system, which acts as a bank of frequency-tuned filters. From the vantage point of a single cochlear filter, in particular a filter sensitive to the stimulus carrier frequency, both AM and FM input correspond to amplitude-modulated output [8]. With respect to FM, this is due to movement of the carrier frequency through the responsive regions of frequency-tuned filters, such that activation strength at a single filter waxes and wanes.

**Figure 1:**
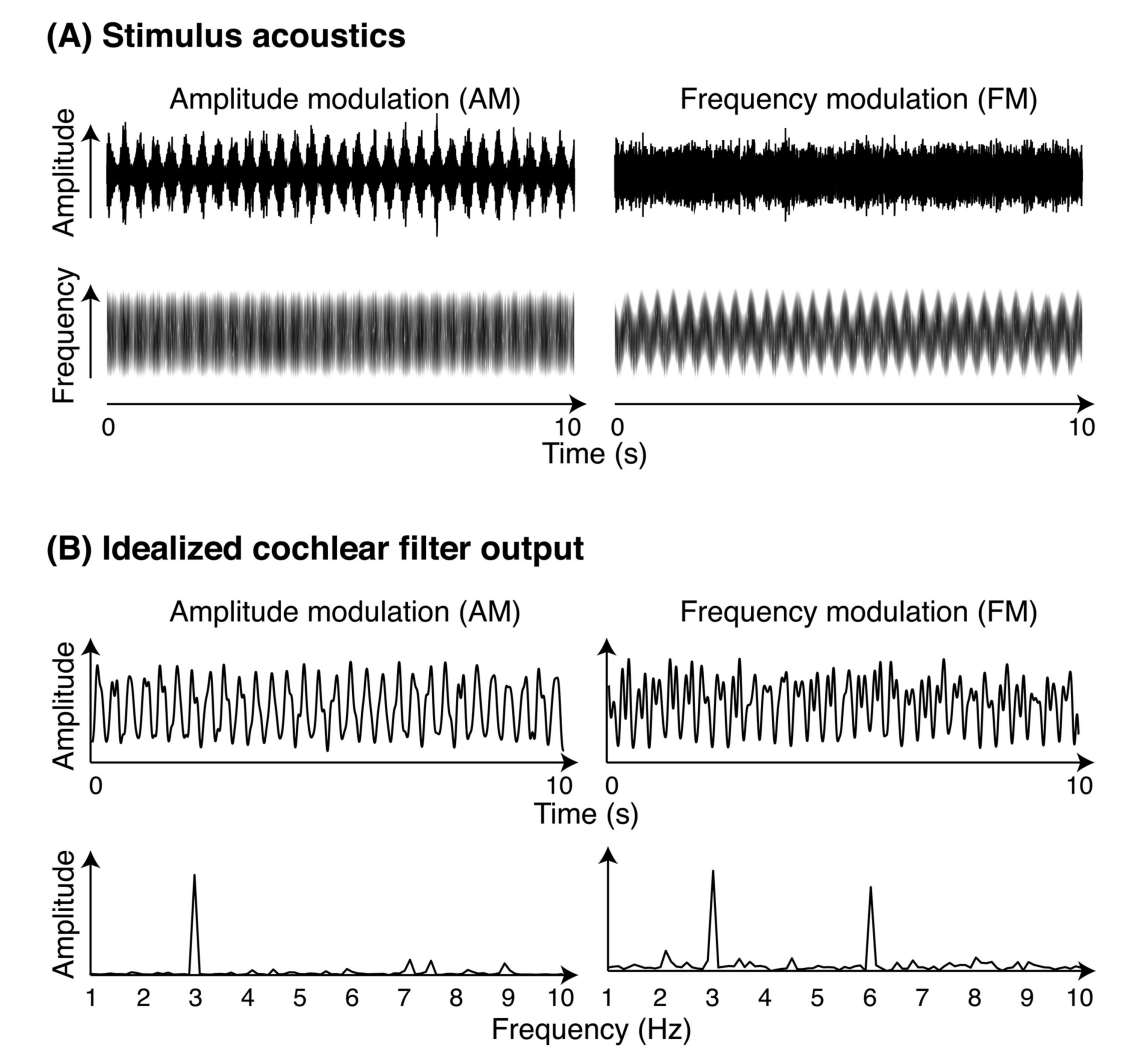
*Stimulus acoustics* and cochlear filter output. (**A**) Amplitude-modulated (AM; left) and frequency-modulated (FM; right) narrow-band noise stimuli. Amplitude-modulated stimuli were characterized by sinusoidal fluctuations in amplitude over time (top left), but a flat frequency profile (bottom left). Frequency-modulated stimuli did not vary systematically in amplitude (top right), but were characterized by sinusoidal fluctuations in frequency over time (bottom right). (**B**) Output from an idealized cochlear-filter model for an exemplary AM (left) and FM (right) stimulus; both exemplary stimuli had 1000-Hz center frequency. The filter output for the AM stimulus was taken from the filter centered on 1125 Hz, and the filter output for the FM stimulus was taken from the filter centered on 875 Hz. Top panels show the amplitude envelope of the filter output, while bottom panels show spectral amplitude as a function of frequency resulting from an FFT.

Figure 1 illustrates this for exemplary AM and FM narrow-band noise stimuli; stimulus acoustics are shown in Figure 1A and details are provided in the Methods section. Figure 1B shows the output of a single filter in response to AM and FM stimulation; details of the idealized cochlear filter model are also provided in the Methods section. There are two features of the cochlear-filter output worth noting. First, the filter output corresponding to both AM and FM stimuli is characterized by amplitude envelopes with dominant modulation in the 3-Hz frequency band. Second, the output corresponding to the FM stimulus is also characterized by power in the 6-Hz frequency band, that is, at the second harmonic of the stimulation frequency. This is because the FM passes through the sensitive region of a single frequency-tuned filter twice per cycle: once during the rising phase and once during the falling phase of the frequency modulation. In general, the amplitude spectra resulting from the FFT on idealized cochlear filter output are consistent with cortical physiological data, and human EEG/MEG data, which we review next.

### Cortical signatures of AM and FM

Single-cell recordings from various sites along the auditory pathway in non-human animals demonstrate that many single units are similarly responsive to AM and FM [9-11]. For example, in a study involving awake marmoset monkeys, Liang and colleagues found that single cells with preferred modulation rates are likely to respond to both AM and FM stimuli with the same modulation frequency, regardless of modulation type. However, the precise firing patterns are notably different for many neurons that fire in a phase-locked manner to the stimulus modulation, that is, neurons that fire at a consistent phase of the stimulus modulation. This is because, at least for auditory cortex neurons with best frequency corresponding to the stimulus carrier frequency, the stimulus frequency passes through the receptive field of the cell twice, once during the rising phase and once during the falling phase of the frequency modulation. For this reason, in order to demonstrate phase-locked firing of such units, Liang and colleagues had to quantify and test stimulus-synchronized discharges at twice the FM frequency, but statistical tests at the modulation frequency were nonsignificant [11]. This result is consistent with the differences in amplitude spectra of cochlear-filter output shown in Figure 1B, where a peak at the second harmonic of the stimulation frequency was observed for FM, but not for AM. To summarize, although individual units responding to AM and FM may be identical in some cases, the time course of unit responses differs between modulation types. 

 In humans, EEG/MEG studies of the auditory steady state response (ASSR, an oscillatory brain response phase locked to periodic auditory stimulation; [12-14]) have also revealed differences between the time courses of responses to AM and FM that are consistent with single-unit data. In particular, frequency-domain representations of responses to FM are characterized by the presence of more and stronger harmonics than responses to AM; the second-harmonic response is markedly larger for FM than AM, in particular at relatively low modulation frequencies [14]. Moreover, ASSR responses to FM are characterized by a shorter phase delay with respect to the stimulus envelope than responses to AM stimulation [14-16]. Notably, shorter phase delays for FM than for AM are predictable in part from a peripheral encoding model based on cochlear-filter output as idealized in Figure 1B. However, predicting the precise AM–FM phase delay from cochlear-filter output is not straightforward because phase delays also reflect filtering effects of the hair cell response system, which we do not address further here [15-17].

### Overview of the current study

In the current human EEG study, we evaluated and compared the amplitude and phase characteristics of AM and FM presented at a slow, speech-relevant rate (3 Hz). Previous human and animal evidence suggests differences in terms of both the spectral amplitude and phase delays of the neural responses estimated from frequency-domain representations. In particular, we expected to observe increased spectral power at the second harmonic of the stimulation frequency and shorter phase delays with respect to the full-band stimulus envelope for FM relative to AM. Moreover, in the current study, we asked whether single-trial time-varying phase or amplitude information would be sufficient to discriminate between neural responses to AM and FM. In this regard, we aimed to characterize the feature(s) of the time-varying neural responses that might support for perceptual separation of AM and FM, and thus allow for the two modulation types to carry independent streams of acoustic information.

## Methods

### Ethics Statement

The procedure was approved of by the ethics committee of the medical faculty of the University of Leipzig and in accordance with the declaration of Helsinki. Written informed consent was obtained from all participants prior to the experiment.

### Participants

Sixteen normal-hearing (self reported), right-handed, native German speakers (8 female; ages 21–31, M = 25.7 yrs, SD = 2.9 yrs) took part in the study. Participants received financial compensation of fifteen €.

### Stimuli

Auditory stimuli were generated by MATLAB software at a sampling rate of 60,000 Hz. Stimuli were 10-s complex tones that were either frequency modulated or amplitude modulated at a rate of 3 Hz. FM depth was 37.5% (Δf/f, where f refers to the carrier frequency and Δf refers to the carrier-to-peak frequency distance), and AM depth was 80% (Figure 1). Modulation depths were calibrated by an experienced listener to be approximately perceptually equal [18]. Complex carrier signals were centered on one of three frequencies (800, 1000, 1200 Hz) and composed of 30 components randomly sampled from a uniform distribution with a 500-Hz range [19]. The amplitude of each component was scaled linearly based on its inverse distance from the center frequency; that is, the center frequency itself was the highest-amplitude component, and component amplitudes decreased with increasing distance from the center frequency. The onset phase of the stimulus was randomized from trial to trial, taking on one of eight values (0, π/4, π/2, 3π/4, π, 5π/4, 3π/2, 7π/4). All stimuli were root mean square (RMS) amplitude-normalized and presented 50 dB above the individual hearing threshold, which was determined prior to experimentation for the AM and FM stimuli presented in the current study.

 Exemplary AM and FM stimuli were processed by an idealized cochlear filter model, One representative stimulus of each type was analyzed by an idealized gamma-tone filter bank [20-22] comprised of nine filters centered on frequencies equally spaced between 0.5 and 1.5 times the center frequency of the narrow-band stimulus (1000 Hz; here, filters were thus centered on frequencies ranging between 500 and 1500 Hz in steps of 125 Hz). In particular, the AM output comes from the filter centered on 1125 Hz, and the FM output comes from the filter centered on 875 Hz. We chose these two filters in line with the suggestion that, due to asymmetry of the cochlear excitation pattern, AM encoding is likely to be largely reliant on filters sensitive to frequencies somewhat higher than the carrier, while FM encoding is likely to rely on filters sensitive to frequencies somewhat lower than the carrier [7,15,23]. The top panels of Figure 1B show the low-pass filtered amplitude envelope of the time-domain cochlear filter output, while the bottom panels show the amplitude spectra as a function of frequency resulting from fast Fourier transforms (FFT) performed on the time-domain output.

### Procedure

The EEG was recorded while participants had the sole task to listen attentively to the stimuli [14,24]. AM and FM stimuli were presented in separate blocks; block order was counterbalanced across participants. Overall, each listener heard 90 FM and 90 AM sounds, for a total of 180 trials. The experiment lasted approximately 90 minutes including preparation of the EEG. 

### Data Acquisition and Analysis

The EEG was recorded from 64 Ag–AgCl electrodes mounted on a custom-made cap (Electro-Cap International), according to the modified and expanded 10–20 system. Signals were recorded continuously with a passband of DC to 200 Hz and digitized at a sampling rate of 500 Hz. The reference electrode was the left mastoid. Bipolar horizontal and vertical electroocculograms (EOGs) were also recorded. Electrode resistance was kept under 5 kΩ. Raw data are available for download online from the Dryad database. 

 All EEG data were analyzed offline using Fieldtrip software (http://www.ru.nl/fcdonders/fieldtrip/; [25]), and custom Matlab (Mathworks, Inc.) scripts. First, continuous EEG data were high-pass filtered at 0.9 Hz. Then, epochs-of-interest were defined as 1.5 seconds preceding to 11.5 seconds following the sound onset in order to capture the response to the full 10-s stimulus. Data were low-pass filtered below 100 Hz, and then artifacts were rejected in two steps. First, independent component analysis (ICA) was used to eliminate blinks, EOG, and muscle activity. This resulted in removal of M = 10.25 ± 4.0 (SD) components in the AM condition and M = 10.0 ± 4.1 (SD) components in the FM condition. Second, individual trials were automatically rejected using a threshold-based rejection routine with a threshold of 120 μV (range). This resulted in removal of an average of 0.56 trials (range 0–4 of 90 trials) for the AM condition and 0.81 trials (range 0–6 of 90 trials) for the FM condition.

#### Frequency-domain analysis

To examine oscillatory brain responses entrained by the 3-Hz stimulation, full-stimulus epochs were analyzed in the frequency domain using a fast Fourier transform (FFT). Time-domain data were multiplied with a Hann window prior to analysis in order to eliminate artifacts due to the assumption of periodic data that is inbuilt in the FFT. Then, amplitude in individual frequency bands was estimated by an FFT conducted on the full stimulus epoch, averaged over trials and after removing the first and final seconds of stimulation in order to eliminate onset- and offset-evoked responses. 

Since the starting phase of the AM and FM stimulation was randomized from trial to trial [19], the FFT was performed twice for each listener. First, before averaging over trials, time-domain brain responses were shifted in time so that either the FM or the AM stimuli would have been perfectly phase locked across trials (“phase-aligned” trials). On the assumption that brain responses were phase locked to the stimulus rhythm with a consistent phase lag across trials, this realignment step was necessary to observe increased amplitude for FFTs calculated on trial-averaged data. Second, spectral amplitude was also calculated without realigning brain responses per trial (“random-phase” trials). Using this technique, each data set for each individual listener acted as its own control.

**Figure 2:**
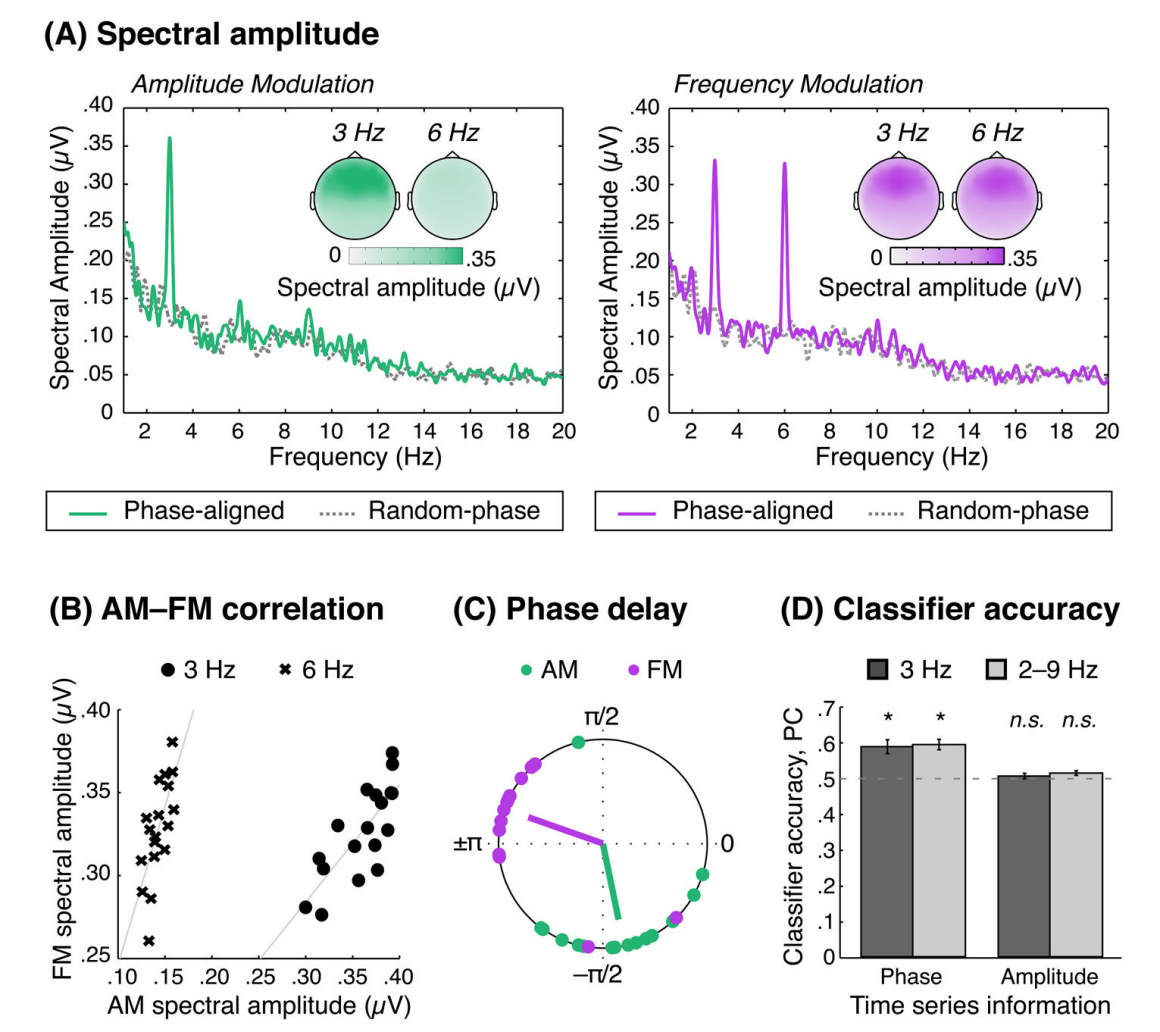
Dissociable neural responses to AM and FM. (**A**) Amplitude spectra as a function of frequency for AM (left, green) and FM (right, purple), averaged over participants. Solid colored lines show amplitude spectra resulting from phase-aligned trials, while dotted gray lines show amplitude spectra resulting from random-phase trials. Amplitudes are averaged over electrodes within an 18-electrode cluster of interest (see Methods). Inserted topographies show spectral amplitude at 3 Hz and 6 Hz. (**B**) FM spectral amplitude for individual participants (averaged over the same electrodes as in A) as a function of AM spectral amplitude, shown for 3 Hz (o’s) and 6 Hz (×’s). (**C**) Phase delays with respect to the full-band stimulus envelope of AM (green) and FM (purple) stimuli. Individual data points correspond to individual participant values, averaged over center frequencies and electrodes. (**D**) Classifier accuracy (proportion of correctly classified trials, PC) for phase time series (left) and amplitude time series (right). Dark gray bars correspond to classification based on only 3-Hz information, while light gray bars correspond to classification based on information in the 2–9 Hz frequency range. The horizontal dotted line corresponds to chance classification performance (PC = .50). Error bars denote standard error of the mean.

Based on examination of spectral amplitude topographies (Figure 2A), we defined an electrode cluster of interest that comprised 18 fronto-central electrode locations: F1, F2, F3, F4, Fz, AF3, AF4, AFz, FC1, FC2, FC3, FC4, FCz, C1, C2, C3, C4, Cz. First, FFTs were averaged over these 18 electrodes, then based on previous examinations of ASSRs to sinusoidal modulation (for a review see 13), we performed hypothesis-directed statistical tests at the stimulation frequency (3 Hz) and the second and third harmonics (6 Hz, 9 Hz, respectively). Separately for the FM and AM conditions, repeated-measures t-tests were performed between the phase-aligned and random-phase data. Moreover, spectral amplitudes for AM vs. FM were compared directly (using phase-aligned data only) with a Modulation Type (AM, FM) × Frequency Band (3 Hz, 6 Hz) repeated-measures ANOVA (to anticipate, spectral amplitude was nonsignificant in the 9 Hz frequency band for both modulation types, and so was not included in the ANOVA). 

Finally, individual differences were investigated by calculating correlations between spectral amplitude values from analyses of AM and FM stimuli separately for the 3-Hz and 6-Hz frequency bands, averaged over the 18 electrodes of interest. Fisher-z transformed correlation coefficients were submitted to a Modulation Type (AM, FM) × Frequency Band (3 Hz, 6 Hz) repeated-measures ANOVA. 

#### Phase-delay analysis

Based on previous work on the ASSR, we suspected that the phase delay of the brain response with respect to the stimulus may have been different for AM vs. FM [15,26]. Thus, phase delays of the neural responses entrained by AM and FM were estimated with respect to the full-band stimulus envelope, where in line with previous work [15,26], we defined the FM delay with respect to the frequency peak. Separately for AM and FM, we constructed three trial-averaged brain responses per listener (one for each center frequency); each average consisted of approximately 30 trials. Time-domain signals were averaged over the 18 electrodes of interest (see previous section). Each average brain response was then submitted to an FFT (details same as above), which yields an estimate of neural phase in each frequency band, where the phase value corresponds to the relative phase of the neural response with respect to the peak of a cosine function. Therefore, phase values resulting from the FFT were subtracted from 2π in order to estimate the delay of the brain signal with respect to the idealized cosine (here, corresponding to the stimulation). Phase-delay values from the 3-Hz frequency band were then submitted to a 2 (Modulation Type) × 3 (Center Frequency) circular ANOVA (*hk test*; [27]). 

#### Single-trial classification of AM and FM

We also pursued a classification-based approach to determine whether single-trial time-varying phase or amplitude information afforded discrimination between neural responses to AM versus FM stimuli [28-30]. The technique we used was as follows. Single-trial time-domain neural responses were submitted to a wavelet convolution in order to generate a time-frequency representation of complex values from which both amplitude and phase information were available. The wavelet convolution was applied at each of the 18 electrodes in our cluster of interest using a time resolution of 30 ms and a frequency resolution of 0.25 Hz. The width of the wavelet increased linearly from 3 cycles at 1 Hz to 8 cycles at 20 Hz. Similar to the frequency-domain analysis described above, we removed the first and final seconds of the neural response in order to avoid influences of onset or offset ERPs on classification performance. Then, we completed the classification twice using phase and amplitude as the features of interest. 

 Phase angles at each time point were estimated from the complex output resulting from the wavelet convolution (using Matlab’s *angle* function). For each trial, we formed two templates – one for responses to AM and one for responses to FM. Templates consisted of the trial-average (circular) mean phase time series in each frequency band of interest. The to-be-classified trial was always left out of the template. For each trial, the phase series was compared against the templates for AM and for FM; as a distance metric, the circular distance between template phase and single-trial phase was calculated and then averaged over time. The template that yielded the smaller mean distance to the single-trial phase series was taken as the predicted category. 

 Similarly, amplitude values per time point were estimated from the same complex output (using Matlab’s *abs* function), and separate templates were formed for AM and FM responses by averaging over trials (in each frequency band and at each electrode of interest), always leaving out the to-be-classified trial. The distance metric between the to-be-classified trial and the template was taken as the absolute value of amplitude differences between time series, summed over time. Again, the template that yielded the smaller distance to the single-trial amplitude series was taken as the predicted category. 

 Classification was performed separately at each electrode within the fronto-central cluster of interest, and then classification accuracies were averaged over electrodes before testing against chance. Moreover, for both phase and amplitude, we completed the classification procedure twice: once for all frequency bands between 2 and 9 Hz, and again for only the 3-Hz frequency band. Paired-samples t-tests (two-tailed) were used to determine whether classifier performance was better when more frequency information was included in the templates. 

 Moreover, in order to assess any bias that might plague the classification approach (i.e., an overall tendency to classify neural responses as AM or FM, respectively), we calculated a signal detection measure of response bias, *c* [31]. For this purpose, “hits” were defined as correct classifications of AM responses as being elicited by AM stimuli, while false alarms were defined as incorrect classifications of FM responses as being elicited by AM stimuli. Then, response bias was calculated according to the standard formula:

c =–1/2*[z(HR) +z(FAR)]

where *HR* corresponds to the proportion of hits and *FAR* corresponds to the proportion of false alarms. Based on the null hypothesis value of no bias (*c* = 0), bias values for each classifier were tested against 0 using a single-sample t-test. In the event of a significant bias result, we also calculated sensitivity for the classifier, *d´*, which is critically independent of bias. Sensitivity was calculated according to the following formula:

d´=z(HR) –z(FAR).

Sensitivity values, where applicable, were then tested against chance performance (*d´*= 0) using a single-sample t-test. 

## Results

### Spectral amplitude

Figure 2A shows amplitude spectra for neural responses to AM stimuli (left, green) and FM stimuli (right, purple), averaged over a fronto-central electrode cluster (see Methods), separately for phase-aligned trials (solid colored lines) and random-phase trials (dashed gray lines). For both AM and FM stimuli, significant peaks in the amplitude spectra (i.e., significant differences between phase-aligned and random-phase trials) were observed in the 3-Hz frequency band (AM: t(15) = 5.89, p < .0001; FM: t(15) = 4.81, p < .0001) and the 6 Hz frequency band (AM: t(15) = 2.52, p = .02; FM: t(15) = 5.47, p < .0001), but not in the 9-Hz frequency band (ps ≥ .10). In order to test for different patterns of results for AM versus FM, a 2 (Frequency: 3 Hz, 6 Hz) × 2 (Stimulus Type: FM, AM) repeated-measures ANOVA was conducted on spectral amplitude values for phase-aligned trials. Critically, the interaction reached significance, F(1,15) = 20.19, p < .0001, due to a significant difference between FM and AM spectral amplitudes in the 6 Hz frequency band (p < .0001), but not in the 3-Hz frequency band (p = .42). 

Figure 2B shows the correlation between individual-participant values of AM spectral amplitude (x-axis) and FM spectral amplitude (y-axis) in the 3-Hz (o’s) and 6-Hz (×’s) frequency bands. In both frequency bands, AM and FM spectral amplitude were highly and significantly correlated (3 Hz: r(30) = .78, p < .0001; 6 Hz: r(30) = .70, p = .001). Note that although error bars are not shown in Figure 2A, Figure 2B shows all individual amplitude values in the relevant frequency bands. 

### Phase delay

Figure 2C shows phase delays for the neural responses (averaged over electrodes in the cluster of interest) with respect to the full-band stimulus envelope. Phase delays are shown separately for AM (green) and FM (purple) stimuli, and are averaged over stimulus center frequencies. A 2 (Modulation Type: AM, FM) × 3 (Center Frequency: 800 Hz, 1000 Hz, 1200 Hz) circular ANOVA revealed a significant main effect of Modulation Type (Χ^2^(2) = 64.63, p < .00001); phase delays for FM stimuli were significantly shorter than phase delays for AM stimuli. Neither the main effect of Center Frequency (Χ^2^(4) = 5.55, p = .24) nor the Modulation Type × Center Frequency interaction (Χ^2^(2) = 0.60, p = .63) reached significance. 

### Single-trial classification of AM and FM

Classification results are shown in Figure 2D. We investigated two separate classifiers that attempted to categorize single-trial neural time series as resulting from AM or FM stimulation using either phase or amplitude information. We also investigated a version of each classifier that relied on only 3-Hz information and one that used information in the 2–9 Hz frequency bands. On the one hand, both versions of the classifier based on phase performed better than chance at classifying the neural signals (3 Hz: t(15) = 4.55, p = .0004; 2–9 Hz: t(15) = 6.80, p = .000006). Moreover, there was no statistically significant difference between accuracy based on which frequency bands were taken into account (t(15) = 0.14, p = .89).

 On the other hand, when operating on amplitude time series data, neither version of the classifier decoded the single-trial neural responses better than chance (3 Hz: t(15) = 0.54, p = .60; 2–9 Hz: t(15) = t(15) = 1.87, p = .08), and classification accuracy for the two classifiers did not differ significantly (t(15) = 0.83, p = .42).

 In order to test whether the success of failure of our classification approach could have been attributable in part to inherent bias, we tested classification biases, *c*, against 0 for all classification conditions. For all but one of the tested classifiers, classification bias did not differ significantly from 0 (*phase*: 3 Hz: *c* = -0.03 ± 0.02 SEM, t(15) = –1.69, p = .11; 2–9 Hz: *c* = 0.03 ± 0.02, t(15) = 1.57, p = .14; *amplitude*: 3 Hz: *c* = 0.018 ± 0.03, t(15) = 0.58, p = .57). The one exception was the classifier based on amplitude time series in the 2–9 Hz frequency bands, where there was a significant tendency to classify neural responses as originating from AM stimulation (*c* = -0.38 ± 0.09, t(15) = –4.50, p = .0004). Nonetheless, after taking into account this classification bias, the amplitude classifier still did not perform significantly better than chance (*d´*= 0.08 ± 0.04, t(15) = 2.06, p = .06).

## Discussion

The current study examined the neural signatures of entrainment to slow (3-Hz) amplitude modulation (AM) and frequency modulation (FM). The main findings were as follows. First, spectral amplitude for FM was higher than for AM in the 6-Hz band, which corresponded to the second harmonic of the stimulation frequency (3 Hz). Second, the entrained neural oscillation lagged the FM stimulus with a shorter phase delay than the AM stimulus. Finally, a classifier successfully predicted which type of stimulus a listener heard on a single trial, based on phase patterns over time. However, a classifier based on amplitude time series was unable to differentiate between neural responses. 

### Spectral amplitude is greater for FM at the second harmonic frequency

We observed similar spectral amplitudes at the stimulation frequency (3 Hz) in response to AM and FM stimuli. However, energy at the second harmonic frequency (6 Hz) was stronger in response to FM stimuli. More energy at the second harmonic for FM than for AM stimuli is predictable from an account of peripheral auditory processing that describes the FM response in terms of the amplitude-modulated cochlear output of a single frequency-tuned filter (Figure 1B). 

 At the cortical level, single-cell recordings from auditory cortex in awake monkeys are also consistent with the results we report here. Specifically, modulation period histograms tend to contain a single peak in response to sinusoidal AM. However, modulation period histograms for transient FM-responsive neurons present either one peak [9,10] or two peaks, with the latter corresponding to neurons that respond each time the stimulus trajectory crosses the center frequency (that is, during the rising phase and during the falling phase of the frequency excursion; [11]). The result is, for FM but not for AM, an energetic peak corresponding to a frequency that is twice that of the modulation. Moreover, in humans, previous research on the ASSR has shown that, especially at low frequencies, FM elicits a strong peak at the second harmonic frequency [13,15,32]. However, second-harmonic responses to AM stimuli have been reported to be much smaller and less consistent than for FM stimuli [14]; see 33 for an exception.

### Phase delay is shorter for FM than for AM

In the current study, AM and FM neural responses differed in terms of their phase delay relative to the broadband stimulus envelope. The phase delay for FM stimuli was consistently shorter than for AM stimuli, in line with previous research on the ASSR [13,15]. It has been suggested that phase-delay differences between responses evoked by AM and FM stimuli are in part attributable to differences in the locations on the basilar membrane where modulation most effectively activates frequency-tuned neurons [15]. Different locations of maximum excitation effectively translate to different travel times for the traveling wave along the basilar membrane, resulting in phase-delay differences. Although our idealized cochlear-filter output (Figure 1B) provided estimates of phase delay for the output of the maximally-excited filter with respect to the full-band stimulus envelope, the prediction of phase relations is further complicated by filtering effects of hair cells in the peripheral auditory system, which are different for AM and FM [15,26]. Thus, we were unable to make quantitative predictions about phase delays based on our idealized cochlear filter model alone. However, the approximate quarter-cycle difference we observed between AM and FM (M = 1.90 radians, 109°) matches values estimated from previous examinations of ASSR at relatively low modulation frequencies [14].

### Classification of AM–FM based on single-trial neural phase information

Our classification approach involved generating templates for neural responses to AM and FM, and then estimating the similarity between each single-trial response and the two templates [28-30]. The template to which the single-trial response was most similar was taken as the predicted stimulus condition. For this approach, we considered phase and amplitude information from the 2–9 Hz frequency bands or from the 3-Hz frequency band only. 

 Regardless of the frequency bands included in the analysis, the classifier based on phase performed significantly above chance. However, regardless of frequency information, the amplitude classifier was unable to differentiate between neural responses to AM and FM. It is perhaps surprising that the 2–9 Hz amplitude classifier was unsuccessful, since the frequency-domain analysis revealed that neural responses to AM versus FM could be differentiated on the basis of spectral amplitude in the 6-Hz frequency band. Analysis of classification bias revealed that the poor performance of the 2–9 Hz amplitude-based classifier could have been due, at least in part, to a bias to classify single-trial neural responses as being associated with an AM stimulus. However, when we examined a bias-free measure of classification accuracy, performance still failed to exceed chance levels. 

 Success of a phase-based classifier and simultaneous failure of an amplitude-based classifier for single-trial neural responses is consistent with a number of recent reports that have taken a similar classification approach for different sets of stimuli [28-30]. All of the cited studies compared classification of single-trial neural responses based on phase versus amplitude information, and all found that time-varying neural phase, but not time-varying amplitude, provided sufficient information for classification. The authors speculated that neural phase “codes for” low-frequency variations in stimulus acoustics. In the current study, in which stimuli were acoustically simple, and single stimuli had constant spectral quality and modulation rate over the whole duration, it is likely that neural responses to AM versus FM were discriminable based entirely on phase-delay differences. In contrast, the referenced studies made use of more complex stimuli with fluctuating spectral content [29], fluctuating modulation rate [30], or consisting of natural speech tokens [28]. In such situations, it is likely that phase delay alone would be insufficient to reconstruct the presented stimulus on single trials, and that more local phase variations effectively “code for” acoustic variations. Future research will be necessary to sort out the precise characteristics of the neural phase time series that correspond to local acoustic variables.

 In sum, the important message to be taken from the current results is that the time-varying phase patterns obtained from human cortical responses allow distinguishing amplitude from frequency modulations. In contrast, human neuroimaging using functional magnetic resonance imaging (fMRI) and source localizations from MEG have revealed substantial overlap in the auditory cortical regions that respond to AM and FM, leading to the suggestion that the two modulation types should necessarily be processed by the same neural machinery [18,34], and in turn calling into question whether independent acoustic information should be transmissible in simultaneous frequency and amplitude modulations applied to a single signal, for instance speech [35]. The present data, and in particular the classifier results, indicate that the problem of separating responses to the two modulation types can be effectively resolved by emphasizing the time-varying, rather than spatial, aspects of the brain signal.

### Relation of the current results to peripheral and central modulation encoding models

It is perhaps instructional to situate the present results within the context of the vast body of psychophysical and neurophysiological work on AM–FM encoding. The primary focus of this work has historically been on whether AM and FM are encoded by the same or different mechanisms, and the appearance of several very recent publications on the topic indicates that this research question has not been unequivocally answered [35,36]. 

 One key finding is that, relative to individual detection thresholds for either AM or FM alone, combining AM and FM within the same stimulus, such that the modulations are out of phase with each other, increases detection thresholds [37-39]. This suggests that the two modulation types cancel, meaning that they must be processed by a unitary mechanism, at least in the auditory periphery. On the other hand, selective adaptation experiments have shown that modulation-detection thresholds were increased following exposure to an adapting stimulus of the same type (i.e., AM–AM, FM–FM; [40,41]), but that detection thresholds did not increase when the adapting stimulus was AM and the test stimulus was FM; the reverse situation (FM–AM) yielded only very small threshold changes. These behavioral data suggest segregated mechanisms underlying AM versus FM encoding. 

 It is notable that discrepancies between conclusions based on such behavioral reports can be largely reconciled by taking into account the modulation rate and carrier frequency of the stimuli. Specifically, combining FM and AM at different phases increases modulation-detection thresholds, specifically for relatively fast modulation rates (>10 Hz) and high carrier frequencies (>6000 Hz; [38,39]). Moreover, selective adaptation for, in particular, FM–AM stimulus pairs is apparent only for modulation rates greater than 8 Hz [40]. In general, at relatively fast modulation rates and at high carrier frequencies, the psychophysical evidence more strongly suggests that FM and AM are coded in the same manner [38,39], whereas for modulation rates below 10 Hz, and in the range of syllable and prosodic variations in speech, AM and FM have been argued to be peripherally encoded by different mechanisms. This conclusion is supported by results indicating that, at low modulation rates, listeners can easily discriminate between AM and FM; however, when the modulation rate is increased, discriminating the two modulation types becomes increasingly difficult [42]. 

 Intriguingly, the picture emerging from auditory cortical examinations is somewhat different. For example, the magnitude of ASSRs recorded during stimulation by a single stimulus containing simultaneous amplitude and frequency modulations can be best predicted by assuming independent contributions of the two responses. That is, in particular at high modulation rates (>40 Hz), there is little attenuation of the ASSR suggesting cancellation by out-of-phase modulations. At relatively low modulation rates (

<~5 Hz) however, single-unit recordings provide strong evidence for a common cortical coding mechanism for temporal modulations more generally [11,34,35,43].

 It is worth noting that the central encoding of fast AM and slow FM has been examined before by studies that concurrently modulated a single carrier stimulus along both dimensions [44-46]. These studies provided evidence for a phase-coding mechanism for slow FM, at least at frequencies below approximately 5 Hz. In particular, the instantaneous carrier frequency (i.e., FM) was coded in terms of the phase delay of the ASSR with respect to the AM with which it was synchronized. However, these studies consistently used AM rates near 40 Hz. Thus, it is difficult to generalize from concurrent fast-AM/slow-FM to non-simultaneous slow AM and FM. 

### Potential effects of modulation depth and rate

The comparison between AM and FM stimuli in the current study involved only one modulation rate (3 Hz) and one modulation depth for each modulation type (80% for AM and 37.5% for FM). Thus, one might speculate on the degree to which our results would be robust to rate and depth manipulations. In this regard, we note that previous work on the ASSR has characterized frequency-domain representations of neural responses to temporal modulation across a range of modulation rates and depths [12-14,32,33,47-51], although direct comparisons of AM and FM are uncommon among the cited studies (exceptions are [14,32]). The general picture that emerges is that, within the range of modulation rates relevant for syllabic and prosodic modulations in human speech (approximately in the delta–theta range; 1–8 Hz), and in which we were interested in the current study, the differences we observed between AM and FM in terms of phase delay and spectral amplitude at the second harmonic frequency would be largely generalizable [14,32,33,50]. Similarly, spectral amplitude and phase delay effects are saturated at the large and salient modulation depths that we investigated here [14,15,51], indicating that modest manipulations to modulation depth would also have been unlikely to affect our results. 

### Implications of dissociable neural signatures of AM and FM for auditory perception

Obleser et al. [19,52] have suggested that the potential role of slow FM as a pacemaker for neural oscillations in the context of speech perception has been overlooked. Indeed, the prosodic contour of speech (and the melodic contour of music) carries time-varying information that is non-redundant with information conveyed by amplitude fluctuations. Supporting the claim that FM may contribute to speech perception above and beyond AM, adding slow FM to degraded speech with an intact amplitude envelope significantly improves speech recognition for both normal-hearing listeners and listeners with cochlear implants [53]. Moreover, cochlear implant (CI) patients in the cited study were able to make use of FM information for speaker recognition and Mandarin tone recognition. These results are fully in line with the logical suggestion of Altman and Gaese [35] that, regardless of any overlap in terms of AM and FM encoding, “AM and FM are two different acoustic parameters that need to be perceptually separated in the process of stimulus recognition for complex signals”. We suggest that independent contributions of slow AM and FM to auditory perception generally, and speech perception more specifically, may be supported by separable neural signatures for the two modulation types [35], which we showed here involves time-varying neural phase information. The current study provides the first direct evidence that single-trial oscillatory cortical responses are sufficient to discriminate between the two modulation types, and thus that these cortical signatures provide an efficient means to dissect simultaneously communicated slow temporal and spectral information in acoustic communication signals.
